# Barriers to and recommendations for take-home naloxone distribution: perspectives from opioid treatment programs in New Mexico

**DOI:** 10.1186/s12954-020-00375-2

**Published:** 2020-05-13

**Authors:** Julie G. Salvador, Andrew L. Sussman, Mikiko Y. Takeda, William G. Katzman, Monica Moya Balasch, Joanna G. Katzman

**Affiliations:** 1 Department of Psychiatry and Behavioral Sciences, University of New Mexico School of Medicine, MSC09 5030 1UNM, Albuquerque, NM 87131-0001 USA; 2 Department of Family and Community Medicine, University of New Mexico School of Medicine, Albuquerque, USA; 3 Department of Pharmacy Practice and Administrative Sciences, College of Pharmacy, University of New Mexico, Albuquerque, USA; 4 University of Michigan, Ann Arbor, USA; 5 Department of Neurosurgery, University of New Mexico School of Medicine, Albuquerque, USA

**Keywords:** Take-home naloxone, Drug policy, Opioid overdose, Opioid treatment programs

## Abstract

**Background:**

Naloxone is a safe and effective medication to help reverse opioid overdose. Providing take-home naloxone to patients in opioid treatment settings is a critical step to reducing opioid overdose deaths. In New Mexico, a US state with one of the highest rates of opioid overdose deaths, legislation was passed in 2017 (House Bill 370) to support take-home naloxone, and followed by naloxone training of Opioid Treatment Program staff to increase distribution.

**Methods:**

Naloxone training was offered to all New Mexico Opioid Treatment Programs along with a baseline survey to assess current practices and barriers to take-home naloxone distribution. Focus groups were conducted approximately 1 year post-training with staff at a subset of the trained Opioid Treatment Programs to assess the impact of the legislation and training provided.

**Results:**

Baseline survey results show most Opioid Treatment Program staff were unfamiliar with House Bill 370, reported conflicting understandings of their agency’s current take-home naloxone practices, and reported a number of barriers at the patient, agency, and policy level. Follow-up focus groups revealed support for House Bill 370 but persistent barriers to its implementation at the patient, agency, and policy level including patient receptivity, cost of naloxone, staff time, and prohibitive pharmacy board regulations.

**Conclusions:**

In spite of targeted legislation and training, provision of take-home naloxone at remained low. This is alarming given the need for this lifesaving medication among the Opioid Treatment Program patient population, and high opioid death rate in New Mexico. Locally, important next steps include clarifying regulatory guidelines and supporting policy/billing changes to offset costs to Opioid Treatment Programs. Globally, additional research is needed to identify the prevalence of take-home naloxone distribution in similar settings, common barriers, and best practices that can be shared to increase access to this vital lifesaving medication in this critical context.

## Introduction

The opioid crisis is a public health emergency worldwide. It is estimated that roughly 27 million people globally have an opioid use disorder (World Health Organization, 2018), with approximately 3 million of those in the USA [1]. In the USA, more than 130 Americans die every day due to unintentional opioid overdose deaths [2]. Opioids accounted for 69.5% of all drug overdose deaths in 2018 [3]. Efforts to address this crisis include expanding access to medications to treat opioid use disorder—including methadone, buprenorphine, and naltrexone—in combination with psychosocial supports. In spite of the research evidence supporting the effectiveness of these medications, there remain barriers to their uptake among behavioral health and primary care settings [4]. Therefore, for many people, treatment for opioid use disorder remains difficult to obtain and access is even more challenging in rural areas [5, 6]. Given this lack of comprehensive treatment, a key harm reduction strategy to reducing opioid overdose deaths is increasing access to naloxone among persons likely to either experience or witness an opioid overdose.

Naloxone is a safe and effective medication that can be easily administered to anyone who is experiencing an opioid overdose and many states have passed laws to help expand access and use of naloxone among laypersons [7]. Methods of naloxone administration include a simple auto injector format (similar in function to an Epi pen for allergic reactions) and a nasal spray. There is strong evidence to support take-home naloxone (THN) in terms of reduction in overdose mortality deaths [8] and the real-world use of naloxone for overdose reversal following adequate training [9, 10]. Federal funding for states to address the opioid use disorder (OUD) epidemic has included a heavy focus on making naloxone and the simple training in its use easily available nationwide. However, access to THN is currently insufficient to address the opioid overdose crisis in the USA [11]. The World Health Organization (WHO) recommends that naloxone be made available to persons likely to witness an opioid overdose, which includes persons at risk of an opioid overdose, their friends, and families [12, 13]. Recent research has demonstrated that providing naloxone to patients in an opioid treatment setting resulted in their use of naloxone to reverse overdose in their community. In fact, nearly 20% of patients provided naloxone used it to reverse an overdose, some even conducting multiple reversals [14].

New Mexico, the setting for the present study, is the fifth largest US state, is predominantly rural and frontier, and has drug overdose death rates consistently higher than the national average [15]. In 2017, New Mexico approved *House Bill 370 Opioid Overdose Education* to help increase access to naloxone within the state’s Opioid Treatment Programs (OTPs) [16]. In New Mexico, OTPs are often referred to as “methadone clinics” but many also provide other medications and treatments for opioid use disorder and substance use disorder more broadly. New Mexico HB 370 mandates that OTPs provide two doses of naloxone and overdose education to each patient, as funding permits. Providing naloxone to OTP patients in New Mexico has shown a positive impact on reducing overdose deaths. A recent study conducted in one New Mexico OTP demonstrated that providing education and naloxone doses to patients resulted in high numbers of opioid overdose reversals [9, 10].

In spite of the known effectiveness of naloxone, its positive safety profile, and global recommendations to make naloxone available to persons likely to witness overdose, there remain significant barriers to implementing this practice at New Mexico’s OTPs. The rationale for the present study was based on the lack of THN distribution among many of New Mexico’s OTPs and examining the impact of New Mexico’s newly passed naloxone legislation combined with comprehensive training of OTP staff on increasing THN distribution in these settings.

## Methods

### Sample and research design

We used a mixed method sequential explanatory design that included a brief baseline survey immediately followed by in-person training in THN (step 1). The second step in the study was to conduct in-person focus groups on current THN practices at the OTP, after each had time to implement THN, approximately 6 to 12 months post-training (step 2). The focus group questions were informed by the baseline survey results and post-training discussion that revealed staff awareness and understanding of New Mexico naloxone legislation, current practice related to naloxone distribution at the OTP, and related challenges (presented in results in Table 1). Focus groups enabled us to collect data regarding current THN practices at the OTP following the training and facilitate discussion to better clarify continued challenges and opportunities for enhanced distribution of THN.

**Table 1:** Opioid Treatment Program naloxone survey results pre-training at baseline

	Study sites								
	*SITE 1	*SITE 2	*SITE 3	*SITE 4	*SITE 5	Site 6	Site 7	Site 8	Total (%)
**Training Participation #**	**13**	**14**	**11**	**5**	**8**	**11**	**5**	**11**	**78**
**Question 1: Are you aware of NM’s HB 370?**									
Yes	2	0	8	1	4	4	0	1	20 (25.6%)
No	10	14	3	4	4	7	5	10	57 (73.1%)
No answer	1								1 (1.3%)
**Question 2: Do you currently prescribe or provide take-home naloxone to your patients? (choose all that apply)**									
Write Rx	2			3	4				9 (10.7%)
Provide take-home		1	9		5				15 (17.8%)
Ask pts to go to pharmacy so that a pharmacist prescribes it	6	2	3		3	2		1	17 (20.3%)
None of the above	5	11	1	2	1	8	5	10	43 (51.2%)
**Question 3: Of the following, which are barriers to dispensing naloxone at your opioid treatment program? (choose all that apply)**									
Affordability	1	2	3	2	1	3	2	3	17 (19.8%)
No mechanism for reimbursement			1		1			2	4 (4.7%)
Unsure what type of naloxone to prescribe (nasal, auto-injector, generic)						1	1		2 (2.3%)
Time to educate patients	2	3		2	2	1	2	2	14 (16.3%)
Patient refusal	1	2	1		5			1	10 (11.6%)
Lack of patient medical history			1					1	2 (2.3%)
Concerns with liability		4				2		3	9 (10.5%)
Inadequate supply	3	1	1		1	3			9 (10.5%)
Belief that it promotes heroin and/or prescription drug abuse							1	1	2 (2.3%)
None of the above*	3	5		1	1	2	1	4	17 (19.8%)
**Focus group participation #**	**6**	**9**	**7**	**5**	**6**	**n/a**	**n/a**	**n/a**	**33**

*Sites in all capitals and underline (SITE 1, SITE 2, etc.) indicate sites that participated in follow-up focus groups to assess THN distribution

#### Step 1: training and survey data collection

The study coordinator contacted all 13 New Mexico OTPs to offer the THN training and schedule the training upon agreement to participate. The coordinator and the lead THN trainer traveled in person to each OTP to conduct the training. The lead trainer is a physician with expertise in THN and New Mexico naloxone guidelines and regulations. Immediately prior to training, all participants were asked to complete a brief three-question survey. Survey questions queried awareness of HB370, current practices regarding provision of naloxone to patients/clients, and barriers to direct naloxone dispensing at the OTP (detailed in Table 1 in “Results” section). Immediately after completing the survey, staff members participated in a comprehensive, in-person training in THN. Each training lasted approximately 1.5–2 h and covered information about New Mexico’s naloxone legislation (HB370), the definition of THN (providing naloxone kits directly to patients at the OTP along with associated education in its use), and training in how naloxone works, how to administer, and specifically what naloxone education to provide to clients. The training was followed by a question and answer period with associated discussion aimed at clarifying any continued misunderstandings about what constitutes THN, identified barriers, and available resources to help address challenges. Resources included where to obtain free naloxone for distribution and contact information for the lead trainer to request any future assistance.

#### Step 2: focus group data collection

We purposefully selected subset of the participating OTPs to participate in an in-person, 1-h semi-structured focus group discussion regarding naloxone distribution focused on awareness/perspectives of HB370, naloxone distribution practices at the OTP, and barriers to distribution. The study coordinator began contacting OTPs to schedule the focus groups 5 months post-training with a goal of completing focus groups approximately 6 to 9 months post-training. We selected OTP sites to participate in the focus groups based on geographic and community variation including north and south locations as well as rural and urban settings. We invited all OTP staff from the selected sites to participate in the focus group discussion. Focus groups were moderated by experienced qualitative researchers on the study team (JS and/or AS). Both JS and AS co-facilitated the first focus group and debriefed afterward to trouble shoot any aspect of the focus group guide. No issues were identified. The following focus groups were conducted independently by either JS or AS. Participants were informed that thematic findings would be aggregated to protect confidentiality. All focus groups were digitally audio-recorded and professionally transcribed.

### Data analysis

Step 1 baseline survey responses were entered into an Excel database and descriptive statistics were used to summarize responses. Focus group data in step 2 were analyzed using a comparative thematic analysis approach aimed at achieving conceptual depth. The lead qualitative researchers (JS and AS) conducted the analysis which consisted of independent reviews of each transcript followed by iterative rounds of refining a coding template based on the major categories of interest included in the focus group guide. Once consensus was reached, all transcripts were fully coded in NVivo 12, a qualitative data analysis software program, and summary reports were generated to facilitate final interpretation and data organization. We focused on each OTP initially as a case study and then conducted comparative analyses to identify potential emergent patterns in the data and develop larger theme categories.

## Results

### Demographics

Table 1 provides details on participation in the baseline survey, training, and follow-up focus groups including the number of participants and time frame for step 1 and step 2 data collection. Eight OTPs agreed to participate in the baseline training and survey. This represented a total of seventy-eight staff. The THN training and surveys were completed with all OTPs between January 11 and May 2, 2018. Five OTPs declined participation reporting that they already distributed THN and therefore did not need the offered training. A wide range of staff participated in the training/survey including top level administration (director/manager levels) and the most frequent roles were addiction counselor (*N* = 37), nurse or nurse practitioner (*N* = 17), and clerk/administrative staff (*N* = 10). Of the eight trained OTPs, five were based in urban settings, while the remainder were located in smaller communities serving rural patients. From among the eight sites that participated in the training and survey, we contacted five to participate in the follow-up focus group. Three of these OTPs were located in urban settings and two in rural settings. A total of 33 staff participated, ranging from a minimum of 5 people to a maximum of 9 at each site. Focus group participants included clinic director/managers and the majority of participants were direct service staff, including nurses and counselors and site administrative personnel. All five focus groups were conducted between October 24, 2018 and March 28, 2019.

### Step 1: survey results

Table 1 shows survey results for the eight OTP sites. Question 1: At the initial training, the majority of OTP staff were not aware of the House Bill 370 requirement to provide two doses of naloxone to patients (73%). Question 2*:* When asked whether the OTP prescribes or provides THN, responses were mixed and some directly contradicted responses from the same OTP site. For example, all eight OTPs had at least one person report that their site did not provide naloxone to patients in any format. However, respondents at three of the sites reported the OTP provided patients with a prescription for naloxone. Staff at six sites reported that they asked patients to go to a pharmacist to obtain naloxone (In New Mexico, pharmacists are required to stock naloxone and provide it to any person upon request). Finally, staff at three sites reported their OTP provided THN. As described below in the focus group results, these conflicting responses are explained by two different interpretations of what constitutes THN. This included an understanding that THN was naloxone distributed directly by OTP staff to patients, and that it could also be an outside agency who comes to the OTP to distribute naloxone to patients during designated times on a weekly or monthly basis. This was clarified during post-training discussion at the OTPs that reported they provided THN and made clear that none of these OTPs were in fact providing THN themselves directly to patients. Question 3: The most commonly reported barriers to providing THN were affordability (22%), time to educate patients (18%), patients refusing naloxone (13%), and concerns with liability (12%). Sites that also participated in the follow-up focus groups are indicated in Table 1 (sites 1–5).

### Step 2: focus groups’ results

#### Agreement with need for THN distribution

At the follow-up focus groups, OTP staff participants widely endorsed providing THN directly to patients. Across the five OTP focus group sites, staff and leadership consistently supported THN to help address the problem of opioid overdose. They believed that most patients were “very receptive” to getting naloxone and that providing training and distribution of naloxone directly by the OTP served to empower patients to take steps to reduce overdose deaths. Respondents were also very supportive of the HB 370 legislation and its implications for OTPs, but again they made it clear at follow-up that the practice of THN was not occurring at the OTPs:

If we can actually give it to them from here, we would know they have it. It would be better.

### Current practice and barriers to THN: patient, OTP system, and policy levels

#### 1) Patient level barriers

Patient naloxone awareness*.* OTP staff felt that patient awareness of naloxone varied from being unaware to fully aware. Lack of awareness meant a patient was less interested in having naloxone. For example, staff noted some patients were unfamiliar with naloxone, including its purpose and the importance of carrying it at all times to reverse an overdose. In these OTPs, providing this education to patients was seen as key to improving receptivity:

A lot of them didn’t want it … but once they got the education, they actually signed [for the medication] saying they wanted it.

In contrast, other comments indicated a high level of education and awareness about naloxone among their patients. In some cases, this seemed to downplay the importance for distribution from the OTP site:

There is nobody in this community that doesn’t know how to get it, and I will say that there’s nobody in the opiate community that doesn’t know how—or somebody to get naloxone from.

Patient receptivity to naloxone. OTP staff reported that some patients did not want to be provided naloxone. This included patients that were actively using and did not want to stay at the OTP long enough to get the short required training on opioid overdose and naloxone use. The fact that naloxone rapidly stopped a “high” was also seen as a deterrent among those still using.

I actually spoke to a patient about naloxone and how I was a big fan of it. He said, “Well, I’m not,” and I said, “Why aren’t you?” He said, “Basically, it pisses off the person who it’s getting distributed to because it ruins the high.”

However, respondents also said that patients who were not using sometimes did not want naloxone because they were “well” and did not need naloxone.

A lot of our patients come in and say, ‘No. I’m here. I’m well. I don’t need naloxone.’ They don’t consider that even “healthy” people can have naloxone and maybe find someone who they knew they’d be able to administer it to.

Burdens of filling prescriptions at pharmacies: logistics, costs and stigma. The broad support for dispensing naloxone at the OTP derives, in part, from a recognition that asking patients to travel to a pharmacy to fill a prescription was a barrier for many, especially in rural areas were transportation was an issue. Staff also cited naloxone cost, either in full or as a co-pay, as a limitation for patients.

I think it would be better [providing THN at OTP], because most of them, we would give the prescription and they wouldn’t go fill it, or they had some kind of issue with their Medicaid. It was always a problem. We gave out over 100 prescriptions, and maybe 20 people filled it.

Finally, staff cited stigma as a barrier to obtaining naloxone from a pharmacy given that some patients feel reluctant to reveal a problem with addiction to pharmacy staff.

That was one of the barriers, too, when we were writing those prescriptions. They didn’t want people to know that they needed that, so a lot of times, it’s pride, embarrassment. This way, it’s here. This is confidential.

Despite the positive views regarding THN, staff acknowledged they mostly provide prescriptions for patients to fill at a pharmacy and/or have an outside agency come to the OTPs to distribute naloxone. Naloxone was not commonly available at OTPs, even for the staff:

We used to keep some …you had two in your drawers, and I had two in mine. We used to keep it just in case. ….We don’t even have any in our desk drawer anymore, because it was outdated.

#### 2) Opioid Treatment Program level barriers

Cost to the Opioid Treatment Program in terms of product purchase and staff time. The most commonly cited critique of the new legislation, House Bill 370 that required that OTPs provided THN, was that the bill did not provide funding for the purchase naloxone. OTP staff said that the high cost of naloxone was a major barrier:

…the thing that concerned me is that there’s no funding for it. I cannot bill Medicaid for naloxone. If they’re requiring us to do it, we should be able to bill it also, because this is pretty expensive.

Staff also discussed the burden associated with THN given limited OTP resources and additional time needed to provide patient training. In response, outside agencies were asked to come to the OTPs to provide naloxone and associated training for patients:

We have too much to do in terms of notes and talking to them (patients) about everything else that we need to do. So, us actually educating them about naloxone is—we wouldn’t be able to add it on to the stuff that we already do.

#### 3) Policy level barriers

Regulations governing naloxone distribution at OTPs. Two of the five sites reported a main barrier to distribution was their understanding of the state’s pharmacy board requirements, which respondents believed mandated that only a pharmacist could distribute naloxone at the OTP. Respondents at these two sites were very clear that this was a key reason why they currently could not distribute naloxone directly.

I’ll tell you, the big thing that we have is the [state pharmacy board], because they require us to distribute it with the clinic licensure, which means any drug that comes out of here…only a doctor or pharmacist can dispense it. Right now, our nurses cannot dispense it at the window when they come in for the methadone.

##### OTP staff suggestions for addressing barriers

During the focus groups, OTP staff presented ideas for addressing barriers regarding THN. Respondents cited the importance of insurance coverage for naloxone and associated training, as mentioned previously, which would facilitate the OTPs’ provision of THN directly to their patients. Another related idea was to make naloxone education a mandatory part of patient’s treatment enabling the training to also be a covered expense:

…it should be mandated, especially with Medicaid patients. If you’re Medicaid … you’ve got to get the training, if you want them to pay for it [your treatment].

OTP staff endorsed a strategy whereby naloxone distribution and associated education would be integrated into patients’ existing OUD treatment and included as part of the package of comprehensive care. This would ensure that financial costs were covered and the patient could obtain naloxone confidentially within the provider-patient relationship.

## Discussion

This study describes the results of a combined policy and training effort in New Mexico to expand THN distribution in the state’s OTPs. The initial survey and training revealed that staff at most sites did not believe their OTP was providing THN. For the three that did report providing THN, it was revealed during the post-training discussion that naloxone was in fact being provided by another agency at the OTP site and on an infrequent basis. Main barriers to providing THN included product cost, staff time, patient reticence, and regulatory concerns. After allowing time to implement THN and to make efforts to address identified barriers, focus groups’ results show that of the five selected OTP sites for follow-up data collection, these sites were still not providing THN, and the barriers they reported at follow-up were similar to those at baseline including cost of product, time for staff to provide the education, patient reticence, and regulatory concerns. The requirement for naloxone combined with training and education of staff about the new legislation and naloxone use was not sufficient for changing practice in these sites. At follow-up, staff were very supportive of THN, but the sites were still implementing other less optimal approaches to provide naloxone to patients, such as giving patients prescriptions, telling them to ask for it from a pharmacy, or in some cases by having an outside agency provide the naloxone to patients at the OTP site.

The findings from this study are important for understanding the usefulness of a policy and training effort to decrease opioid overdose death rates through naloxone distribution in a high-need population. The most important finding from this study is that among the OTPs who participated in our initial survey, training and follow-up focus groups, the barriers they initially identified persisted at follow-up. Despite delivery of a detailed training featuring information about resources for obtaining free naloxone, access to additional assistance, and the statewide policy requiring THN be provided (HB 370), the OTPs in our focus group sample did not report the practice change outcomes that we anticipated. Given national and local focus on addressing the opioid crisis, and in particular reducing overdose deaths, these results are concerning. Our findings highlight the need to more substantively address foundational barriers, such as cost of product and staff time, to support THN distribution by OTP providers. In the context of addressing these policy and system-level challenges, it is also important to promote strategies that reduce social stigma and protect confidentiality.

Furthermore, it is important to note that the barriers we identified in this study are not unique to the New Mexico OTP context and suggests that addressing these identified barriers is critical broadly (nationally and internationally) for increasing access to naloxone for those who need it the most—those at risk for witnessing overdose which includes persons in opioid use disorder treatment. For example, the fact that persons with OUD sometimes do not want naloxone has been identified elsewhere [17, 18]. Negative views of naloxone by persons with OUD, particularly that it can precipitate withdrawal and be associated with police involvement is also well known [19–21]. The need for increased patient education is consistent with findings from a recent study on THN in Baltimore, MD, which found that among participants who had access to THN or who witnessed an overdose, additional training such as “booster” sessions to enhance skills following initial education were important [22]. Recent research with persons who use opioids also found poor knowledge about naloxone among this high-need population [23]. Among the New Mexico OTP staff, the importance of education was underscored by the suggestion that naloxone training be a mandatory part of treatment. This is a creative policy change that would help OTP staff provide this education to patients who may be reluctant and also ties the cost of the naloxone kits and education into the full practice of OUD treatment. Another major barrier is naloxone cost. Currently, treatment of OUD using FDA approved medications is a billable service, but the provision of naloxone and associated education is not. Concerns with the high cost of naloxone have also been documented in other studies and commentaries and are a critical limitation to expansion of THN [17, 24]. In fact, because naloxone is costly, there are strong policy arguments that call for focused distribution to persons most likely to benefit, which includes persons who are likely to witness overdose, including persons who use illicit opioids, their family, and friends [24]. In our study, even in a highly focused target population, naloxone cost is a barrier to getting this lifesaving product in the hands of those most in need. The suggestion to tie OUD treatment with the provision of naloxone and associated education is an important policy strategy to tackle both patient reluctance and product cost barriers. Outside of the OTP context solely, broad efforts to make naloxone free and easily available to all persons likely to witness overdose show positive results. For example, research has estimated that the comprehensive THN program implemented in British Columbia resulted in hundreds of averted deaths and that earlier implementation would have further saved lives [25].

Finally, respondents in our study also reported there were regulatory guidelines that prohibited the distribution of THN, for example, by requiring that only a pharmacist or physician could perform provide naloxone to patients. The baseline training provided at each site made it clear that THN distribution was not in conflict with any regulations, and in fact a pharmacist or doctor did not need to be the sole distributor to patients. Interestingly, Winstanley et al. also found a similar lack of clarity regarding policies pertaining to the handling and management of naloxone kits [17]. Given the fact at direct education did not alter this as documented in our focus groups, and given that other studies also show confusion around naloxone regulations, we recommend use of memorandums of agreement/understanding. This can ensure that key players, such as agencies, regulatory boards, state’s opioid treatment authorities (SOTAs), and similar have written documentation of the regulations and the commitment of agencies to provide THN, as recommended.

### Limitations and future research

We conducted this study to examine the impact of a practice improvement effort combining policy and training to enhance naloxone distribution among New Mexico OTPs. Sites that agreed to participate in the survey, training, and focus groups may be different from the OTPs who did not participate, so our findings cannot be used to generalize about practice at all OTPs, in New Mexico or elsewhere. While not designed to be broadly generalizable, our findings remain instructive about barriers to THN distribution and contribute to the limited literature specific to the practice of THN in the critical context of opioid treatment programs. Given the recommendation by the World Health Organization that persons most likely to witness an opioid overdose have access to naloxone (2018), we believe it is evident that additional research is needed to determine THN practices in other opioid treatment settings, common barriers faced, and best practices that can be shared to improve implementation of this lifesaving practice.

## Conclusions

In conclusion, our study found persistent barriers to THN distribution that echo findings from the naloxone literature. Given evidence on the use of naloxone among OTP patients to reverse opioid overdoses in their communities [9] and the fact that the kinds of barriers identified in this study are widespread and persistent, this study is important for highlighting the need to tackle these barriers directly and swiftly. This includes ensuring regulatory guidelines governing naloxone distribution are clarified among OTPs and identifying and implementing effective models to support implementation of THN. At the OTP level, the focus of the present study, this includes financial and policy related changes that will cover the cost of naloxone kits and associated patient training.

## Data Availability

The datasets used and/or analyzed during the current study are available from the corresponding author on reasonable request.

## Abbreviations

OUD: Opioid use disorder
THN: Take-home naloxone
OTP: Opioid treatment program
